# Distance-based clustering using QUBO formulations

**DOI:** 10.1038/s41598-022-06559-z

**Published:** 2022-02-17

**Authors:** Nasa Matsumoto, Yohei Hamakawa, Kosuke Tatsumura, Kazue Kudo

**Affiliations:** 1 Department of Computer Science, Ochanomizu University, Tokyo, 112-8610 Japan; 2 Corporate Research and Development Center, Toshiba Corporation, Kawasaki, 212-8582 Japan; 3 Graduate School of Information Sciences, Tohoku University, Sendai, 980-8579 Japan

**Keywords:** Condensed-matter physics, Statistical physics, thermodynamics and nonlinear dynamics

## Abstract

In computer science, clustering is a technique for grouping data. Ising machines can solve distance-based clustering problems described by quadratic unconstrained binary optimization (QUBO) formulations. A typical simple method using an Ising machine makes each cluster size equal and is not suitable for clustering unevenly distributed data. We propose a new clustering method that provides better performance than the simple method, especially for unevenly distributed data. The proposed method is a hybrid algorithm including an iterative process that comprises solving a discrete optimization problem with an Ising machine and calculating parameters with a general-purpose computer. To minimize the communication overhead between the Ising machine and the general-purpose computer, we employed a low-latency Ising machine implementing the simulated bifurcation algorithm with a field-programmable gate array attached to a local server. The proposed method results in clustering 200 unevenly distributed data points with a clustering score 18% higher than that of the simple method. The discrete optimization with 2000 variables is performed 100 times per iteration, and the overhead time is reduced to approximately 20% of the total execution time. These results suggest that hybrid algorithms using Ising machines can efficiently solve practical optimization problems.

## Introduction

Many combinatorial optimization problems can be described by the Ising model or quadratic unconstrained binary optimization (QUBO) formulations^1^. Ising machines, which are special-purpose computers for solving combinatorial optimization problems, have attracted significant interest in recent years. Inspired by the first quantum annealer^2^, several devices have been developed, such as digital processors based on simulated annealing (SA)^3–9^, those on simulated bifurcation (SB)^10–12^, coherent Ising machines implemented with pulsed lasers^13–18^, and other types of optical Ising machines^19–21^. Most Ising machines accept an objective function in the form of a Hamiltonian formulated by the Ising model or QUBO formulation. Ising machines return binary solutions that minimize the objective function, although they do not always return optimal solutions because of their heuristic nature. Recent research on the application of Ising machines has shifted from simple combinatorial optimization to hybrid methods using both an Ising machine and a general-purpose computer^22–27^. Most hybrid methods are iterative methods that offload the sampling or combinatorial optimization step to an Ising machine.

Clustering is a technique for grouping data such that the members in each cluster have similar characteristics. Although there are various types of clustering, this work focuses on non-hierarchical and distance-based clustering. Because clustering can be formulated as a combinatorial optimization problem, several algorithms using Ising machines have been developed^28–36^. Clustering methods using a quantum computer and a quantum annealer have also been proposed and investigated^37,38^. A typical simple method using an Ising machine minimizes the distances between data points in the same cluster, resulting in cluster sizes that are approximately equal^34,35^. Let us suppose that a small group is away from other large groups. Then, using this method, part of a large group is merged into a small group such that the number of members in each group becomes almost equal. This implies that this method is not suitable for clustering unevenly distributed data.

In this work, we propose a clustering method using an Ising machine that applies to unevenly distributed data. We compare the clustering of two data sets, one uniformly distributed and the other unevenly distributed, using the simple method and the proposed method. Employing the average silhouette coefficient, we evaluate clustering performance. The proposed method provides better clustering results than the simple method, especially for unevenly distributed data. The proposed method is a hybrid algorithm that solves discrete optimization problems iteratively. The discrete optimization described in a QUBO formulation is performed by an Ising machine, while a general-purpose (conventional) computer calculates parameters for the QUBO formulation in each iteration.

The proposed method could be implemented in one of three possible ways: (i) discrete optimization executed on a remote Ising machine provided as a cloud service, (ii) discrete optimization undertaken using a local Ising machine, and (iii) all computations undertaken on a local general-purpose computer. The first approach incurs a high communication cost with the Ising machine, and so is unsuitable for hybrid algorithms. The second implementation option offloads the computation of discrete optimization steps to the accelerator attached to a local server. This approach has the benefit of a much lower communication cost with the Ising machine than the first approach. In this work, we compare the second and third implementations. The Ising machine used is an SB-based machine implemented with a field-programmable gate array (FPGA)^12^. The low latency of the SB-based machine is advantageous for executing the proposed method. Because the method requires the iterative computation of discrete optimization, a low communication cost between an Ising machine and a general-purpose computer is essential for high performance.

This work demonstrates that the proposed hybrid method using an Ising machine provides high-quality results in clustering problems. The method’s effectiveness suggests a new usage that takes advantage of a low-latency Ising machine. Moreover, this work will highly motivate developing iterative hybrid algorithms. Although hybrid algorithms using an Ising machine and a general-purpose computer take extra time for communication, they have many practical applications. The reduction in communication cost enables iterative hybrid algorithms to solve practical optimization problems efficiently. The proposed method will also be applicable as a quantum-classical hybrid algorithm when the communication cost reduces significantly.

## Related work

We briefly review some recent works on the clustering method using Ising machines and quantum computers. Simple distance-based clustering is a typical example using an Ising machine or a quantum computer. Simple clustering methods based on the distance between data points^34,35,37^ and graph partitioning^33^ using a quantum annealer still attract some interest. Several methods inspired by classical clustering have been proposed and developed recently. For example, quantum-assisted clustering based on similarities^28^, K-means^29–31^ and K-Medoids^32^ clustering on a quantum annealer, as well as K-means clustering on a gate-model quantum computer^38^, have been proposed. K-means-like clustering on a digital Ising machine has also been investigated^36^.

## Models and algorithms

We examine two clustering methods. One is the typical simple method in which the Hamiltonian is given as

1 $$\begin{aligned} H = \sum _{g=1}^G \sum _{i<j} d_{ij} x_{i,g}x_{j,g} + \alpha \sum _{i=1}^N \left( \sum _{g=1}^G x_{i,g} - 1 \right) ^2, \end{aligned}$$

where $d_{ij}$ is the distance between points *i* and *j*, *N* is the number of points, and *G* is the number of groups. The sum $\sum _{i<j}$ is taken over all combinations that satisfy $1\le i<j \le N$. Here, we refer to this method as the simple-cost method. When point *i* belongs to group *g*, $x_{i,g}=1$; otherwise, $x_{i,g}=0$. The first term of the Hamiltonian sums up all the distances between point pairs in each group. The second term of the Hamiltonian requires each point to belong to exactly one group, and $\alpha$ is a positive constant. This term gives a penalty when a point belongs to more than two groups or does not belong to any group. Because the number of point pairs in each group dominates in the Hamiltonian, each group tends to have almost the same number of points.

To resolve the issue, we propose another Hamiltonian described by

2 $$\begin{aligned} H=\sum _{g=1}^G\frac{\sum _{i<j} d_{ij}x_{i,g}x_{j,g}}{N_g(N_g-1)} + \alpha \sum _{i=1}^N \left( \sum _{g=1}^G x_{i,g} - 1 \right) ^2, \end{aligned}$$

where $N_g=\sum _{i=1}^N x_{i,g}$ is the number of points in group *g*. The number of nonzero terms in $\sum _{i<j} d_{ij}x_{i,g}x_{j,g}$ equals the number of point pairs in group *g*, i.e., $N_g(N_g-1)/2$. Therefore, $\sum _{i<j} d_{ij}x_{i,g}x_{j,g}/[N_g(N_g-1)]$ is the average distance between the point pairs in group *g*, where a factor of 2 is omitted for simplicity. In other words, the first term on the right-hand side of Eq. (2) expresses the sum of the average distances between the point pairs in each group. The dominance of the number of point pairs in each group in Eq. (1) is dissolved in Eq. (2). It will be experimentally shown later (in Fig. 4) that this formulation works well, especially for unevenly distributed data points.

Ising machines cannot directly minimize non-QUBO formulations such as Eq. (2). Instead, we employ a hybrid algorithm in which another Hamiltonian described by a QUBO formulation is iteratively minimized. We refer to this method as the iterative fractional-cost method.

The iterative fractional-cost method originates from the hybrid parametric method proposed to solve the vehicle routing problem^24^. The hybrid parametric method is an extension of an inexact parametric algorithm to solve fractional programming problems^39^. Instead of minimizing the original fractional objective function, the hybrid method iteratively solves the corresponding parametric problem in the discrete-optimization step, using a quantum annealer or an Ising machine.

The algorithm of the iterative fractional-cost method is as follows.

1. Set the error parameter $\delta$, the iteration counter *n* as $n=0$, and parameter $\lambda$ as an initial value $\lambda _0=0$.
2. Minimize the following Hamiltonian with an Ising machine.
  3 $$\begin{aligned} H = \sum _{g=1}^G \left[ \sum _{i<j}^N d_{ij} x_{i,g} x_{j,g} -\lambda _n \left( \sum _{i=1}^N x_{i,g}\right) \left( \sum _{i=1}^N x_{i,g}-1\right) \right] + \alpha \sum _{i=1}^N\left( \sum _{g=1}^G x_{i,g} - 1 \right) ^2, \end{aligned}$$
  where $\{x_{i,g}\}$ represents binary variables. Let $\{\hat{x}_{i,g}\}$ denote an obtained solution.
3. If $\{\hat{x}_{i,g}\}$ is a feasible solution, set $\lambda$ for the next iteration as
  4 $$\begin{aligned} \lambda _{n+1} = \sum _{g=1}^G \frac{\sum _{i<j} d_{ij}\hat{x}_{i,g}\hat{x}_{j,g}}{\hat{N}_g(\hat{N}_g-1)}, \end{aligned}$$
  where $\hat{N}_g=\sum _i \hat{x}_{i,g}$. Otherwise, terminate with no solution.
4. If $|\lambda _{n+1} -\lambda _n| \le \delta$, terminate with $\{\hat{x}_{i,g}\}$ as the final solution. Otherwise, return to step 2 with $n\rightarrow n+1$ while $n+1 < n_\mathrm{max}$. Alternatively, terminate with no solution when $n+1=n_\mathrm{max}$.

If the algorithm successfully terminates, $\lambda _{n+1}$ is expected to give the minimum cost. Because of the heuristic nature of the Ising machine, $\lambda _{n+1}$ does not always coincide with the global optimal solution. However, it is proved that the inexact parametric method converges to a global optimum if the obtained solution at the discrete-optimization step (step 2) is a good approximation^39^.

The key point of the iterative fractional-cost method is the use of an Ising machine to minimize Eq. (3), which is a QUBO formulation, instead of the fractional-cost Hamiltonian, Eq. (2). In the simulation below, we take the error parameter as $\delta =10^{-6}$ and the maximum iteration number as $n_\mathrm{max}=10$.

## Results

We apply the two methods to two kinds of data sets. One is the set of points distributed uniformly in a square region. The other is the set of unevenly distributed points in the same size region. Each set comprises 200 points ($N=200$), and we take the number of groups as $G=10$ here, so that there are 2,000 binary variables in total. The data sets are provided as Supplementary Information.

### Simple-cost method

**Figure 1:**
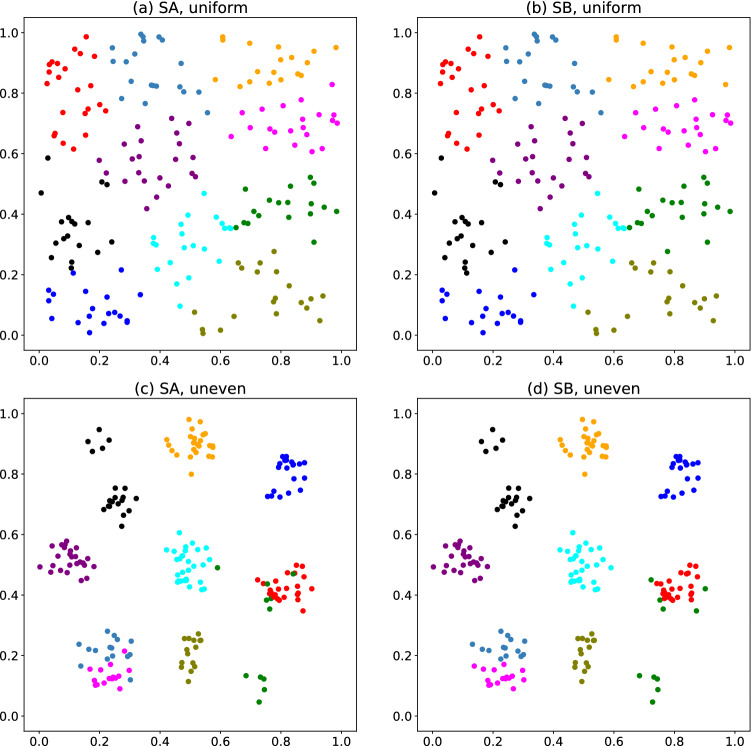
Clustering results for (**a**,**b**) uniformly and (**c**,**d**) unevenly distributed data. The color of each point represents its group. (**a**,**c**) were obtained using SA, whereas (**b**,**d**) were obtained using SB.

In the simple-cost method, we minimize Eq. (1) using the SA or SB. Figure 1 exhibits clustering results, where points with the same color belong to the same group. Each panel shows a result selected to demonstrate the best performance of the method. We obtained Fig. 1a,c using SA, and Fig. 1b,d using SB. We find almost no difference between Fig. 1a and Fig. 1b, with little difference between Fig. 1c and Fig. 1d. For the unevenly distributed data, an apparent cluster is divided into two groups, and some points in apparent clusters are classified as other group members.

**Figure 2:**
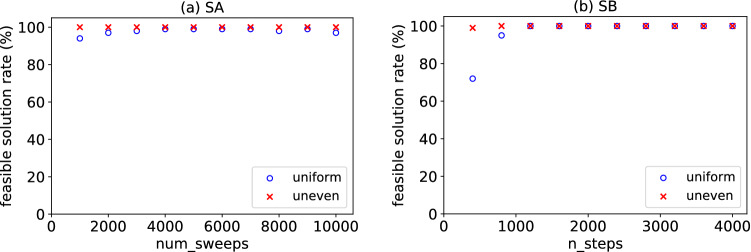
Feasible solution rates in (**a**) SA and (**b**) SB. The horizontal axis is the parameter representing the number of time steps: (**a**) the number of annealing steps, (**b**) the number of SB time steps. $\circ$ and $\times$ are for the data sets with uniform and uneven distributions, respectively.

Solutions provided by SA or SB are not always valid because they sometimes violate the constraint that each point should belong to exactly one group. We call the solution satisfying this constraint a feasible solution. Here, we perform the simulation 100 times for each data set, using either SA or SB for the given time steps. The ratio of feasible solutions among the solutions obtained in the 100 trials is the feasible solution rate shown in Fig. 2. The horizontal axis in Fig. 2 represents the number of time steps. The larger the number of steps, the slower the SA or SB process. Here, the hyperparameter values are $\alpha =5.5$ and $\alpha =6.0$ for SA and SB, respectively, for the data set with a uniform distribution; $\alpha =5$ for SA and SB for that with an uneven distribution. In Fig. 2, the feasible solution rate is almost 100%, except for the region where the number of steps is relatively small. However, a low feasible solution rate does not always result in an inferior result. By changing the value of $\alpha$, we obtained similar clustering results even though feasible solution rates were 40–80%.

**Figure 3:**
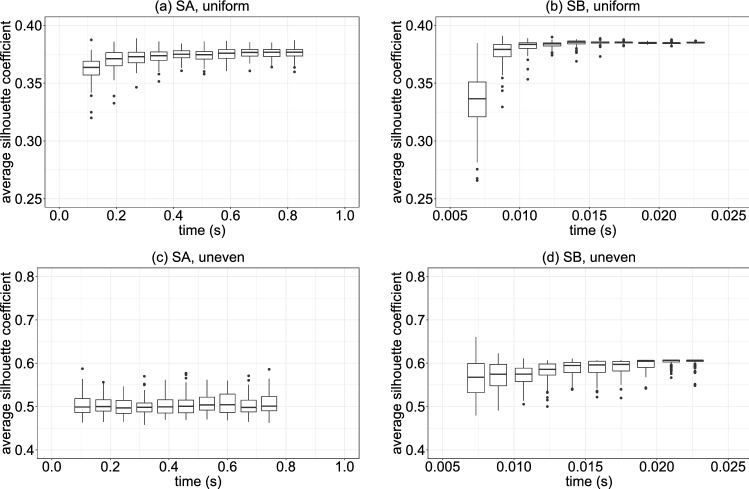
Box plots of the average silhouette coefficient for (**a**,**b**) the uniformly distributed data set and (**c**,**d**) the unevenly distributed one. (**a**,**c**) were obtained using SA, whereas (**b**,**d**) were obtained using SB. The horizontal axis is execution time.

For evaluation of the clustering result, we introduce the silhouette coefficient^40^. Because the silhouette coefficient is defined for each data point, we take the average of every point’s silhouette coefficient in a feasible solution. The better the clustering result, the higher the average silhouette coefficient. Figure 3 demonstrates how clustering results depend on the execution time. The box plots show the distribution of average silhouette coefficients for feasible solutions. The execution time is the average of the feasible solutions for each number of steps. The average silhouette coefficient is about 0.35–0.39 or lower for the uniformly distributed data set [Fig. 3a,b], while it is approximately 0.45–0.6 or higher for the unevenly distributed one [Fig. 3c,d]. The difference reflects the difference in data distribution. On the other hand, the difference between SA and SB is significant. Although the execution time is much shorter for SB than for SA, the average silhouette coefficient for SB is almost equal or even higher. Moreover, the variance of the average silhouette coefficient is small for SB in the long-time region.

### Iterative fractional-cost method

**Figure 4:**
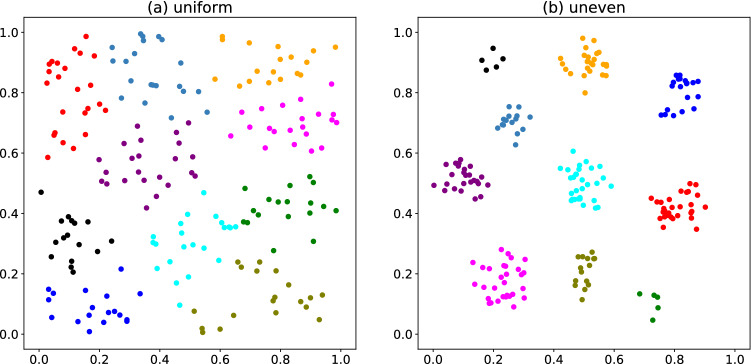
Clustering results obtained using SB for the (**a**) uniformly and (**b**) unevenly distributed data.

We use only SB to perform discrete optimization in the iterative fractional-cost method. Because the simulation using SA had a longer run time and provided similar or worse results than SB in the simple-cost method, we cannot expect SA to provide better results than SB in the iterative fractional-cost method. Figure 4 exhibits examples of clustering results in the iterative fractional-cost method. Compared with Fig. 1, unevenly distributed data points are well classified.

**Figure 5:**
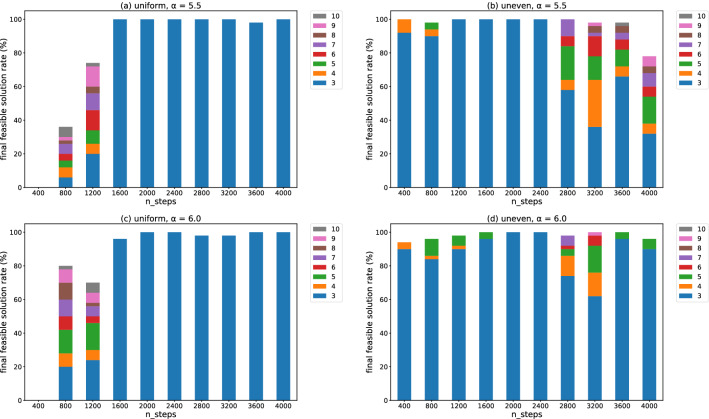
Final feasible solution rates for (**a**,**c**) the uniformly distributed data set and (**b**,**d**) the unevenly distributed one. The number in the legend is the number of iterations of steps 2–4 in the iterative fractional-cost method. The horizontal axis represents the number of SB time steps. The hyperparameters $\alpha =5.5$ in (**a**,**b**), and $\alpha =6.0$ in (**c**,**d**).

Figure 5 illustrates the final feasible solution rates. Here, the simulation was run 50 times for each data set. In other words, the final feasible solution rate is the ratio of feasible solutions obtained at the end of the algorithm among the 50 trials. The number in the legend represents the number of iterations of steps 2–4 in the iterative fractional-cost method before the final solution is obtained. In this work, we perform optimization sampling 100 times at the discrete optimization step (step 2) to obtain several feasible solutions for Eq. (3). The optimal solution among them is $\hat{\varvec{x}}$, which evaluates $\lambda _{n+1}$ in Eq. (4). Even if $\hat{\varvec{x}}$ is feasible at each iteration, this method sometimes fails to obtain a final solution within $n_\mathrm{max}=10$.

In most cases in Fig. 5, the final feasible solutions are obtained in three iterations. More iterations are required to reach a final feasible solution in the small-number-of-step region for the uniformly distributed data, as shown in Fig. 5a,c. The simulation for the unevenly distributed data requires more iterations for a large number of steps, as shown in Fig. 5b,d. The discrete optimization step works better when the number of steps is large compared to when it is small, which can cause overfitting. If the solution is overfitted to a temporal parameter $\lambda _n$ in each iteration, it may become difficult for the algorithm to converge. However, controlling the hyperparameter $\alpha$ can result in the simulation requiring fewer iterations.

**Figure 6:**
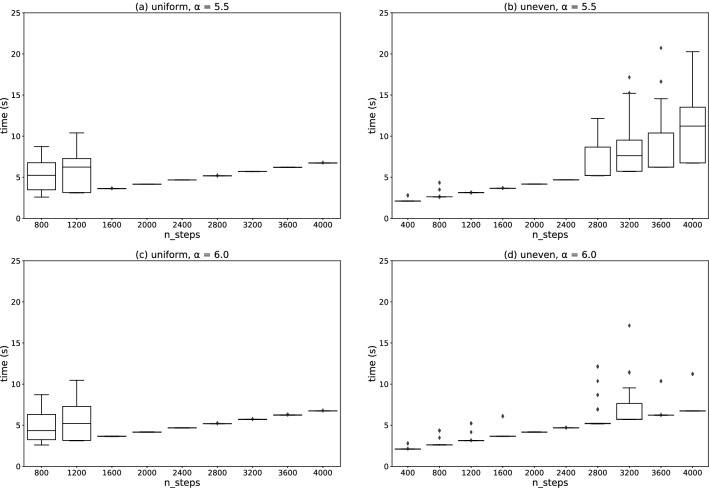
Box plots of execution time for (**a**,**c**) uniformly and (**b**,**d**) unevenly distributed data. The horizontal axis represents the number of SB time steps. The hyperparameters $\alpha =5.5$ in (**a**,**b**), and $\alpha =6.0$ in (**c**,**d**).

Figure 6 exhibits the execution time taken for the discrete optimization in the iterative fractional-cost method. Each panel of the figure corresponds to each one of Fig. 5. The execution time depends on the number of iterations, optimization samplings at the discrete optimization step, and SB time steps.

The average silhouette coefficient is almost independent of $\alpha$ and the number of SB time steps in the region corresponding to Figs. 5 and 6. For the uniformly distributed data, the average silhouette coefficient is 0.392–0.394, which is comparable to that of the simple-cost method. However, for the unevenly distributed data, the average silhouette coefficient is 0.709–0.716, approximately 18% higher than that of the simple-cost method.

## Discussion

In this work, we have compared two clustering methods based on QUBO formulations. One is the simple-cost method, and the other is the iterative fractional-cost method. We have applied each method to data sets with uniform and uneven distributions. The simulation of the simple-cost method is performed using SA and SB, whereas that of the iterative fractional-cost method is performed using only SB.

Clustering results highly depend on data distribution. For an uneven distribution, the iterative fractional-cost method works better than the simple-cost method. However, the iterative fractional-cost method requires more time to obtain a result because it requires several iterations. If the variation in cluster size is significant, the iterative fractional-cost method is a better choice. Otherwise, the simple-cost method is acceptable.

The difference between SA and SB is significant, especially in execution time. The difference in execution time arises mainly from the parallelization of an algorithm. In this work, SA is based on the single spin-flip Monte Carlo method and is out of parallelization. However, SB is executed through massively parallel processing. The parallelization is the key to the acceleration of finding reasonable solutions.

Another reason why SB outperformed SA in execution time is that the accelerator made of an FPGA is attached to the local server. Even if a remote Ising machine or a quantum annealer solves QUBO problems faster than SA, the communication time between the machine and the local server may cancel the acceleration. The results shown above imply that the acceleration by the SB-based machine overcame the communication overhead between the accelerator and the general-purpose processor, i.e., CPU, in the local server.

This work paves the way for solving practical optimization problems requiring iterative hybrid algorithms in a reasonable time by using an Ising machine. For most present Ising machines, including quantum annealers, iterative hybrid algorithms are relatively inefficient, mainly because of communication overheads. However, iterative hybrid methods using a high-speed Ising machine with low latency can provide high performance in solving practical optimization problems. The present work demonstrates such an example.

## Methods

### Execution time

In this section, we define one-shot execution time as the time required to solve a discrete optimization problem. The detailed definition differs between SA and SB because different architectures were used for each method. For SA, we used the dwave-neal package^41^, which is an SA software, and measured the time from calling the SA sampler to obtaining solutions. When the SA sampler executes SA $n_\mathrm{reads}$ times per call, the one-shot execution time is the measured time divided by $n_\mathrm{reads}$. In SB, the one-shot execution time, which is measured for each call, includes the FPGA calculation time and the communication time between the CPU and FPGA.

**Figure 7:**
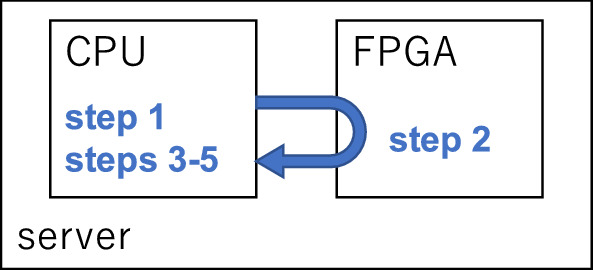
Schematics of the iterative fractional-cost method using SB. Only the discrete optimization step (step 2), in which the QUBO Hamiltonian in Eq. (3) is minimized, is executed in the FPGA.

The execution time for the simple-cost method (shown in Fig. 3) is the same as the one-shot execution time. On the other hand, in the iterative fractional-cost method, the execution time shown in Fig. 6 is defined as the sum of the one-shot execution times. As shown in Fig. 7, only the discrete optimization step (step 2) is executed in the FPGA. The step is iterated several times, and 100 shots of SB are executed at each iteration. Thus, the execution time for the iterative fractional-cost method is several hundred times longer than that for the simple-cost method.

The overhead time in SB is a minor component of execution time. For example, as shown in Fig. 3b, the execution time for 2000 SB time steps is approximately 14 ms. The communication time between the CPU and FPGA, which is independent of the number of SB time steps, is approximately 3 ms. Thus, on average, the overhead time is approximately 20% of the execution time for the problem considered in this work.

### Ballistic simulated bifurcation

The SB algorithm, which is a quantum-inspired algorithm, was proposed to accelerate combinatorial optimization^10–12^. The SB is based on adiabatic evolution in classical nonlinear systems showing bifurcations. The SB algorithm finds a spin configuration minimizing the Ising model energy defined by

5 $$\begin{aligned} E = -\frac{1}{2}\sum _{i,j=1}^N J_{i,j} s_i s_j + \sum _{i=1}^N h_i s_j, \end{aligned}$$

where $s_i=\pm 1$ is the *i*th spin, *N* is the number of spins, $J_{i,j}$ is the coupling coefficient between the *i*th and *j*th spins, and $h_i$ is the local field on the *i*th spin.

SB has several variants, the first of which was adiabatic SB (aSB). The set of equations for aSB is given by

6 $$\begin{aligned} \frac{dx_i}{dt}&= a_0y_i, \end{aligned}$$

7 $$\begin{aligned} \frac{dy_i}{dt}&= -x_i^3 -\left[ a_0-a(t)\right] x_i -\eta h_i + c_0\sum _{j=1}^NJ_{i,j}x_j, \end{aligned}$$

where $x_i$ and $y_i$ are real numbers corresponding to the *i*th spin, $a_0$, $c_0$ and $\eta$ are positive constants, and *a*(*t*) is a control parameter that increases from zero to $a_0$. Equations (6) and (7) express the equations of motion of the classical particle corresponding to the *i*th spin: $x_i$ and $y_i$ represent the position and momentum of the *i*th particle, respectively. Classical particles interact in a potential whose shape gradually changes. The sign of $x_i$ at the end of the time evolution gives $s_i$ as the solution to the problem described by Eq. (5). Because $x_i$ is a continuous variable, even though spin $s_i=\pm 1$ is discrete, analog errors arise in the aSB.

The ballistic SB used in this work was developed to suppress analog errors^12^. Instead of the nonlinear term $x_i^3$ in Eq. (7), perfectly inelastic walls are introduced at $x_i=\pm 1$. Using the symplectic Euler method, we numerically solve the set of equations of motion for bSB, whose updating rule is as follows^12^.

8 $$\begin{aligned} y_i(t_{k+1})&= y_i(t_k) + \left\{ -[a_0-a(t_k)]x_i(t_k) -\eta h_i + c_0\sum _{j=1}^NJ_{i,j}x_j(t_k)\right\} \Delta _t, \end{aligned}$$

9 $$\begin{aligned} x_i(t_{k+1})&= x_i(t_k) + a_0 y_i(t_{k+1})\Delta _t, \end{aligned}$$

where $t_k$ is the *k*th time step, and $\Delta _t$ is the time-step width. Namely, $t_{k+1}=t_k+\Delta _t$. Here, we take $t_0=0$. At each time after the update of $x_i$, if $|x_i|>1$, we replace $x_i$ with $\mathrm {sgn}(x_i)=\pm 1$ and set $y_i=0$.

### Parameters

The hyperparameter $\alpha$ in Eqs. (1) and (3) are tuned such that the feasible solution rate and average silhouette coefficient become large. Setting the number of time steps as 10,000 and 2,000 for SA and SB, respectively, we changed $\alpha$ from 3 to 7 in 0.5 increments. The feasible solution rate (final feasible solution rate in the iterative fractional-cost method) rapidly increases at a specific value of $\alpha$. We selected one from a few $\alpha$ values around the value based on the following criteria: (i) The average values of the average silhouette coefficients are higher than those of any other $\alpha$ in the entire time-step region of our experiments. (ii) If the average values are similar among the $\alpha$ values, the (final) feasible solution rates are high.

In SA, the parameter controlling the annealing time is the number of sweeps or steps, called num_sweeps in the dwave-neal package. In SB, the number of steps $n_\mathrm{steps}$ controls the increasing rate of *a*(*t*), that is, $a(t_k)=(1-k/n_\mathrm{steps})a_0$. In this work, we set $c_0=a_0/\lambda _\mathrm{max}$, where $\lambda _\mathrm{max}$ is the maximum eigenvalue of matrix $J_{ij}$, and $a_0=\eta =1$ and $\Delta _t=0.5$ in Eqs. (8) and (9).

### Average silhouette coefficient

The silhouette coefficient is defined for each data point, and it is higher when the point belongs to a well-defined cluster. The silhouette coefficient for point *i* is defined by

10 $$\begin{aligned} s(i) = \frac{b(i)-a(i)}{\max (a(i), b(i))}, \end{aligned}$$

where *a*(*i*) is the mean distance between point *i* and the other points in the same group, and *b*(*i*) is the mean distance between point *i* and points in the neighboring group. However, if a point belongs to several groups or does not belong to any group, the silhouette coefficient cannot be properly calculated. Therefore, we calculate the silhouette coefficient for a feasible solution in which each point belongs to exactly one group and take the average over all the points in the feasible solution.
